# Benefit Design and Access to Dental Care Among Seniors With Medicare Advantage Dental Benefits

**DOI:** 10.1001/jamahealthforum.2024.5123

**Published:** 2025-01-24

**Authors:** Kamyar Nasseh, Astha Singhal, Marko Vujicic, Lisa Simon

**Affiliations:** 1 American Dental Association Health Policy Institute, Chicago, Illinois; 2 College of Dentistry, University of Florida, Gainesville; 3 Division of General Internal Medicine and Primary Care, Brigham and Women’s Hospital, Boston, Massachusetts

## Abstract

**Importance:**

Although Medicare Advantage plans frequently offer dental benefits, enrollees report lower rates of dental care use and higher rates of unmet dental need compared with individuals with employer-sponsored benefits. It is unknown which attributes of Medicare Advantage dental plans are associated with enhanced dental care access.

**Objective:**

To determine attributes of Medicare Advantage dental plans associated with higher rates of dental care use and lower rates of unmet dental need.

**Design, Setting, and Participants:**

This cross-sectional study included respondents from the 2019 Medicare Current Beneficiary Survey whose Medicare Advantage plan identifiers were linked to 2019 Medicare Advantage dental plan data from the Centers for Medicare & Medicaid Services. Respondents enrolled in a Medicare Advantage dental plan for all 12 months in 2019. Data analysis was performed between May and August 2024.

**Exposures:**

Medicare Advantage plans offering dental benefits.

**Main Outcomes and Measures:**

Main outcomes were unmet dental need in the past year, unmet dental need due to cost in the past year, and whether the respondent visited a dentist in the past year. Outcomes were measured in a survey of individuals ages 65 years and older.

**Results:**

In a sample including up to 1789 enrollees (mean [SD] age, 74.7 [7.4] years; 58.4% female; and 13.2% lived in a rural county), enrollees in Medicare Advantage HMO plans were 7.0 percentage points (95% CI, 3.2 to 10.9 percentage points) more likely to report unmet dental need and 4.4 percentage points (95% CI, 0.9 to 7.8 percentage points) more likely to report an unmet dental need due to cost. Prior authorization was associated with an increase of 4.5 percentage points (95% CI, 0.3 to 8.7 percentage points) in unmet dental need. Relative to plans that imposed no out-of-pocket costs on comprehensive services, plans that covered only preventive services were associated with an increase of 12.1 percentage points (95% CI, 3.2 to 21.0 percentage points) in unmet dental need and an increase of 7.8 percentage points (95% CI, 0.6 to 15.0 percentage points) in unmet dental need due to cost. Relative to plans with up to a $500 annual plan maximum, benefits with no annual plan maximum were associated with a decrease of −12.4 percentage points (95% CI, −20.9 to −3.8 percentage points) in unmet dental need.

**Conclusions and Relevance:**

This study found that restrictive characteristics of Medicare Advantage dental plans are associated with greater unmet dental need and financial barriers to care. Results of this study suggest that increasing annual plan maximums or eliminating them entirely from benefit plans could decrease unmet dental need and increase dental care utilization.

## Key Points

**Question:**

What attributes of Medicare Advantage (MA) dental benefits are associated with lower rates of unmet dental need and higher rates of dental care utilization among enrollees?

**Findings:**

In this cross-sectional study of 1789 MA enrollees with 12 months of dental benefits, variations in dental plan benefit attributes were associated with differences in unmet dental need, financial barriers to dental care, and dental care use. Plans with higher annual plan maximums and plans that impose no annual maximums were associated with lower rates of unmet dental need and financial barriers to dental care and higher rates of dental care use.

**Meaning:**

Results of this study suggest that less restrictive plan benefit design may be associated with better access to dental care and lower rates of unmet dental needs among MA enrollees.

## Teaser

This cross-sectional study assesses attributes of Medicare Advantage dental plans associated with higher rates of dental care use and lower rates of unmet dental need.

## Introduction

Oral health is a critical component of healthy aging.^1^ Yet many older US residents face affordability challenges when it comes to going to the dentist. In fact, cost barriers for dental care are more severe than for any other type of health care service.^2,3^ Part of the reason is that traditional Medicare (TM) does not cover dental services except for patients in need of medically necessary procedures such as tooth extractions to treat mouth infections prior to cancer treatment.^4^ The landscape is more complex when we look at how Medicare Advantage (MA) plans address dental care. While MA enrollment has grown, with more than half of Medicare beneficiaries enrolling in MA as of 2023,^5^ the percentage of MA plans offering dental coverage has also increased. From 2020 to 2024, the percentage of MA plans offering coverage for preventive dental care services (eg, checkups, cleanings) increased from 75% to 90% while those offering comprehensive dental care services (eg, restorations, root canals) increased from 50% to 85% of plans.^6^ Racial and ethnic minority groups, along with those with low educational attainment and lower incomes, are especially likely to enroll in MA plans with dental benefits, suggesting that these benefits could play a role in reducing inequities in access to dental care services and, ultimately, oral health.^7,8^

However, evidence suggests that having dental coverage via MA has little impact on dental outcomes. MA enrollees still have difficulty accessing dental care compared with other populations. Among US residents ages 65 years and older, 12.6% of enrollees with an MA dental benefit reported a cost barrier to dental care compared with 7.4% with non-MA private dental insurance.^9^ This suggests that MA dental coverage may not provide the same financial protection as private dental insurance. Another study also shows that enrollment in MA does not result in higher dental care use compared with TM once people become eligible for Medicare after age 65 years.^10^ Similarly, compared with TM enrollees, MA enrollees experience a larger decrease in dental spending after transitioning into Medicare from private dental insurance after retirement.^11^ Enrollees with MA dental benefits also experience substantial out-of-pocket costs for dental care, nearly equivalent to TM enrollees.^12^ These results may suggest that MA dental benefit design may be insufficient when it comes to reducing financial barriers to dental care among Medicare enrollees. In this study, we examine the characteristics of MA dental benefit plans an their association with unmet dental needs, financial barriers to dental care, and dental care use.

## Methods

### Data Sources

We examined a cross-section of beneficiaries with MA dental benefits from the 2019 Medicare Current Beneficiary Survey (MCBS). Data analysis was performed between May and August 2024. We followed the Strengthening the Reporting of Observational Studies in Epidemiology (STROBE) reporting guideline. The University of Florida Institutional Review Board determined this study to be exempt, as it uses publicly available data sources without Health Insurance Portability and Accountability Act identifiers. The MCBS is a nationally representative survey of individuals enrolled in TM or MA. The survey uses a rotating cohort design that samples approximately 15 000 enrollees per year. Each year, the response rate to the baseline interview is about 60%; in subsequent interviews, the response rate exceeds 80%.^13^ We focused on the respondents who completed the cost supplement, from which measures of dental care utilization were derived.

The MCBS documents monthly MA contract and plan identifiers for each beneficiary. We linked MA dental plan characteristics to each MCBS respondent using the respondent’s county of residence, MA contract number, and MA plan number. We extracted MA dental plan data from publicly available 2019 Plan Benefit Package files published by the Centers for Medicare & Medicaid Services (CMS) and released quarterly. The Plan Benefit Package files include information on plan coverage and supplemental benefits (eg, dental, vision, hearing) for all MA payers that submit a bid to CMS. These files include detailed information on the services covered under an MA dental plan (eg, restorations, radiographs, oral examinations), general plan characteristics (eg, health maintenance organization [HMO] vs preferred provider organization, prior authorization requirements, referral requirements), and benefit design attributes (eg, coinsurance/copayment levels, plan deductibles, annual plan benefit maximums).^14^

### Sample

**Figure 1.**
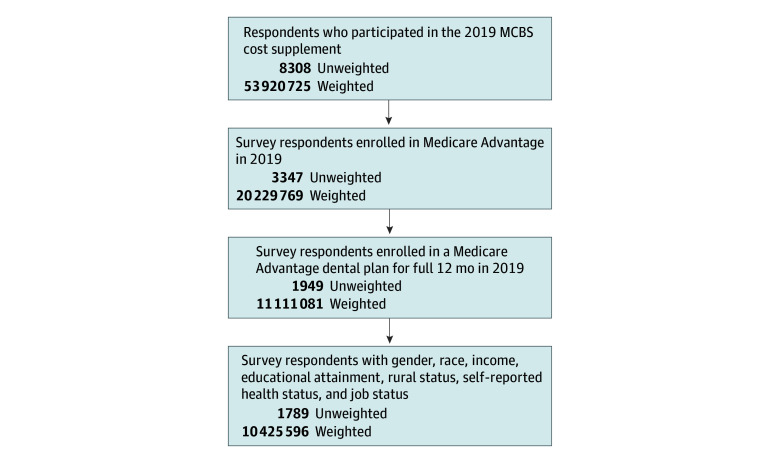
Sample Selection Flowchart MCBS indicates Medicare Current Beneficiary Survey.

We analyzed data from respondents who participated in the 2019 MCBS cost supplement (unweighted N = 8308; weighted N = 53 920 725). We further restricted our sample to respondents whose MA contract and plan identifiers were able to be matched to identical MA contract and identifiers in the Plan Benefit Package files. For each quarter of 2019, we restricted the sample to only MA plans with coverage for preventive or comprehensive dental services. After merging these data and removing MCBS respondents not enrolled in an MA dental plan for the full year, our sample resulted in 1949 unweighted and 11 111 081 weighted observations. We considered an MCBS respondent to be enrolled in an MA dental plan if that plan offered any preventive dental services (radiographs, oral examinations, prophylaxis, or fluoride treatment) or any comprehensive dental services (nonroutine services, diagnostics, restorative, endodontics, periodontics, prosthodontics, tooth extractions, or oral and maxillofacial surgery). After removing observations with missing gender, race, income, educational attainment, rural status, self-reported health status, or job status, our final analytic sample contained 1789 unweighted or 10 425 596 weighted observations (Figure 1). The factors are used as covariates in the regression analysis.

### Dependent Variables

Our outcomes were 3 reported measures of dental care access: (1) whether the survey respondent visited a dentist in the past year; (2) whether the survey respondent reported an unmet dental need in the past year; and (3) whether the survey respondent reported an unmet dental need due to cost in the past year. Unmet dental need due to cost is subset of overall unmet dental need. We examined these 3 outcomes as dependent variables in our analysis.

### Plan Characteristic Covariates

In our analysis, we examined several dental plan characteristics and benefit design covariates. The plan characteristics in our regression models included whether it was an HMO dental plan, whether the dental plan covered at least two dental cleanings in a year, whether the dental plan required prior authorization for any dental services, whether the plan required a referral, and whether all typical dental services were covered by the plan (radiographs, examinations, dental cleanings, diagnostics, restorations, endodontics, periodontics, prosthodontics, tooth extractions, and oral and maxillofacial surgery).

Benefit design covariates in our regression models included whether the respondent was enrolled in an MA dental plan mandating out-of-pocket (OOP) payments for preventive services (either coinsurance or copayments), a categorical variable classifying OOP costs for comprehensive dental services (no OOP, positive copayment, coinsurance less than 50%, coinsurance greater than or equal to 50%, or enrolled in a preventive-only plan) and a categorical variable for annual plan benefit maximum (less than $500, between $501 and $1500, between $1501 and $2000, between $2001 and $2500, greater than $2500, or no annual plan benefit maximum).

### Statistical Analysis

We estimated probit regression models controlling for individual and county-level covariates to assess the association between MA dental plan attributes and dental care utilization, unmet dental need, and unmet dental need due to cost. As individual-level covariates, we included sex (male or female), race and ethnicity (Asian, Black, Hispanic, White, or other race [no further information is available]), household income as a percentage of the federal poverty level (FPL) (less than or equal to 100% of FPL, between 101% and less than or equal to 135% of FPL, between 136% and less than or equal to 200% of FPL, and more than 200% of FPL), age (65-74, 75-84, ≥85 years), educational attainment (less than high school, high school, some college, college, postgraduate), census region (New England/Middle-Atlantic, East North Central, West North Central, South Atlantic, East South Central, West South Central, Mountain, Pacific), Medicare-Medicaid dual eligibility status, job status (a binary variable for whether the respondent or spouse was working), self-reported health (good/excellent vs poor/fair health), and rural/urban residence determined by Rural-Urban Commuting Area Codes. County-level covariates included November 2019 MA penetration by quantile (percentage of Medicare-eligible individuals enrolled in MA),^15^ the log of 2018 median annual household income, percentage in poverty in 2018, population density based on the 2010 census, and the number of dentists per 100 000 population. Apart from MA penetration, other county-level covariates were extracted from the Area Health Resource File.^16^ For ease of interpretability, after probit estimation, we calculated marginal effect estimates to express differences in terms of percentage points.

We performed all analyses in Stata SE, version 18.0 (StataCorp LLC). In all analyses, we applied survey weights from the 2019 MCBS cost supplement and accounted for the MCBS complex survey design. Our threshold for statistical significance was set at *P* < .05 and all hypothesis tests were 2-sided.

## Results

### Comparison of Dental Care Access by Insurance Status

There were 1789 MA enrollees with 12 months of dental benefits. Respondents enrolled in an MA dental plan have lower dental care access compared with those enrolled in an MA plan without a dental benefit or those enrolled in traditional Medicare (eTable 1 in Supplement 1). Compared with TM enrollees, MA enrollees with a dental benefit had higher rates of unmet dental need (12.5% [95% CI, 10.5%-14.4%] for MA enrollees vs 8.1% [95% CI, 6.9%-9.3%] for TM enrollees) and lower rates of dental care use (47.0% [95% CI, 44.4%-49.6%] for MA enrollees vs 59.2% [57.4%-61.0%] for TM enrollees).

### Sample Characteristics

Of 1789 enrollees, respondents had a mean (SD) age of 74.7 (7.4) years, were 58.4% female, and 13.2% lived in a rural county. Beneficiaries identified themselves as Asian (1.9%), Black (15.6%), Hispanic (13.4%), White (67.8%), or another race (1.3%), and 20.1% of MA dental beneficiaries had a household income at or below the FPL. Twenty-two percent of MA dental beneficiaries had less than a high school education, 29.4% had a high school degree, 27.9% had some college, 12.5% had a college degree, and 8.4% had a postgraduate degree. In our analytic sample, 25.5% of beneficiaries were dual eligible and 80.1% reported good or excellent general health (eTable 2 in Supplement 1).

In our sample (Table 1), 12.7% of respondents reported an unmet dental need, 9.5% reported an unmet dental need due to cost, and 49.2% visited a dentist in the year. Among MA beneficiaries with a dental benefit, 70.8% enrolled in an HMO dental plan, 90.3% were in plans that offered at least two dental cleanings per year, 75.0% had OOP costs for preventive services, 27.5% were in plans that required a referral, 59.2% were in plans that required a prior authorization, and 29.8% were in plans that offered a full spectrum of dental services. Regarding OOP costs for comprehensive dental services, 34.1% were in plans with no OOP costs, 11% of enrollees were in plans that required a greater than $0 copayment, 4.5% were in plans with coinsurance between 0% and 50%, 24.9% were in a plan with at least 50% coinsurance, 25.6% were in a dental plan that did not cover comprehensive services. Regarding annual plan maximums, 9.9% of enrollees were in plans with between $0 and $500 maximums, 24.6% were in plans with between $501 and $1500 maximums, 14.8% were in plans with between $1501 and $2000 maximums, 4.8% were in plans with between $2001 and $2500 maximums, 3.0% were in dental plan with greater than $2500 maximum, and 42.9% faced no benefit maximum.

**Table 1.** Summary Statistics, Distribution of Dental Outcomes, and Medicare Advantage Dental Plan Characteristics From 1789 Observations

Characteristic	Weighted % (95% CI)
Dental access and utilization	
Reported unmet dental need^a^	12.7 (10.5-14.8)
Reported financial barrier to dental care^a^	9.5 (7.7-11.2)
Dental visit	49.2 (46.4-52.0)
Plan characteristics	
HMO plan	70.8 (68.3-73.3)
≥2 Dental cleanings per year	90.3 (88.8-91.9)
OOP cost for preventive services	75.0 (72.7-77.4)
Referral required	27.5 (25.0-30.1)
Prior authorization required	59.2 (56.4-61.9)
All dental services offered	29.8 (27.2-32.3)
OOP cost for comprehensive services, $	
0 Cost	34.1 (31.5-36.8)
>0 Copayment	11.0 (9.2-12.8)
0% <Coinsurance <50%	4.5 (3.4-5.5)
≥50% Coinsurance	24.9 (22.5-27.3)
Preventive-only benefit	25.6 (23.1-28.1)
Annual benefit maximum, $	
0 <Benefit maximum ≤500	9.9 (8.3-11.6)
500 <Benefit maximum ≤1500	24.6 (22.2-27.0)
1500 <Benefit maximum ≤2000	14.8 (12.7-16.9)
2000 <Benefit maximum ≤2500	4.8 (3.7-5.8)
Benefit maximum >2500	3.0 (2.2-3.8)
No benefit maximum	42.9 (40.1-45.6)

Abbreviations: HMO, health maintenance organization; OOP, out of pocket; PPO, preferred provider organization.

^a^ Based on analytic sample of 1455 observations (weighted N = 8.3 million) due to missing observations on unmet dental need and unmet dental need to due to cost. All estimates are weighted and consider the complex survey design of the Medicare Current Beneficiary Survey.

### Multivariable Models

**Figure 2.**
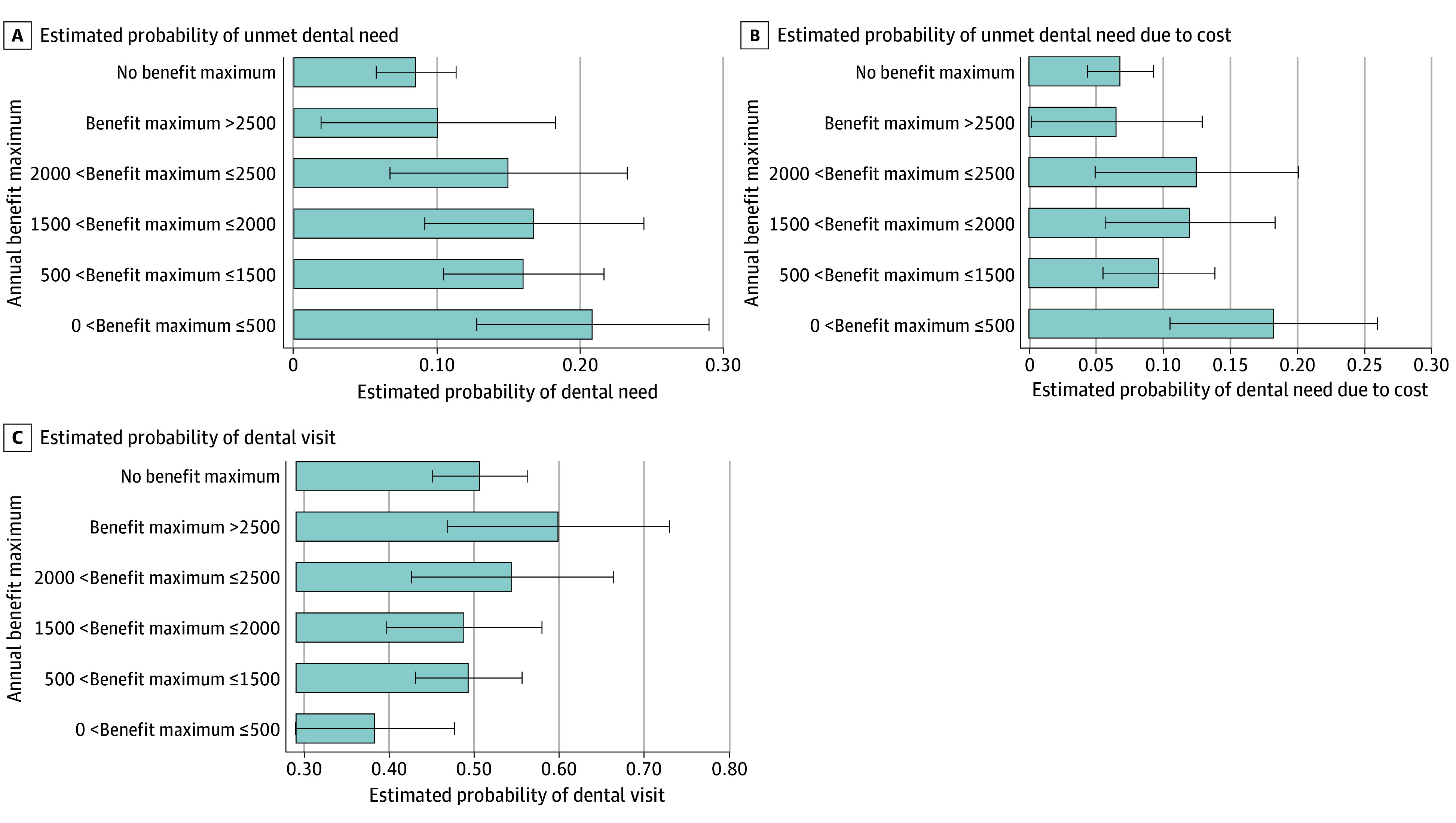
Adjusted Estimated Probability of Unmet Dental Need, Unmet Dental Need Due to Cost, and Dental Care Use by Annual Benefit Maximum Levels Benefit levels are in $ US. Whiskers indicate 95% CIs.

MA beneficiaries enrolled in an HMO dental plan were 7.0 percentage points (95% CI, 3.2-10.9 percentage points; *P* < .001) more likely to report an unmet dental need compared with individuals not in HMO dental plan. The percentage point difference for individuals enrolled in plans that do not impose OOP payments on preventive services compared with individuals enrolled in plans that do not impose OOP payments on preventive services was 3.9 percentage points (95% CI, −0.5 to 8.3 percentage points; *P* = .08), although the finding was not statistically significant. Prior authorization requirements were also associated with unmet dental need (4.5 percentage points [95% CI, 0.3-8.7 percentage points]; *P* = .03). Compared with plans with no OOP costs for comprehensive services, plans not covering those services were associated with unmet dental need (12.1 percentage points [95% CI, 3.2-21.0 percentage points]; *P* = .008). Otherwise, at different positive levels of coinsurance or copayment levels for comprehensive services, there was no substantial variation in reported unmet dental need among MA dental plan enrollees. Relative to plans that imposed up to a $500 annual plan maximum, MA enrollees in plans with no annual maximum reported lower rates of unmet dental need (−12.4 percentage points [95% CI, −20.9 to −3.8 percentage points]; *P* = .004) (Table 2). The estimated probability of an MA enrollee with dental benefits reporting an unmet dental need increased as annual plan maximums decreased (Figure 2A).

**Table 2.** Association of Unmet Dental Need, Unmet Dental Need Due to Cost, and Dental Care Utilization With Medicare Advantage Dental Plan Attributes^a^

Dependent variable	Percentage point, mean (SE) [95% CI]		
	Reported unmet dental need (n=1455 observations)	Reported unmet dental need due to cost (n=1455 observations	Visited dentist in year (=1789 observations)
HMO plan	7.0 (2.0) [3.2 to 10.9]^b^	4.4 (1.8) [0.9 to 7.8]^c^	−2.4 (3.6) [−9.5 to 4.7]
≥2 Dental cleanings per year	0.4 (3.4) [−6.2 to 7.0]	0.1 (3.3) [−6.3 to 6.4]	−4.8 (4.7) [−14.0 to 4.3]
OOP for preventive services	3.9 (2.2) [−0.5 to 8.3]^d^	2.0 (1.9) [−1.7 to 5.8]	−1.1 (3.5) [−8.0 to 5.8]
Referral required	4.0 (3.2) [−2.3 to 10.2]	4.3 (3.0) [−1.5 to 10.2]	3.4 (4.0) [−4.5 to 11.3]
Prior authorization required	4.5 (2.1) [0.3 to 8.7]^c^	3.2 (1.8) [−0.4 to 6.8]^d^	−2.8 (3.3) [−9.2 to 3.6]
All dental services offered	3.2 (2.8) [−2.3 to 8.7]	2.0 (2.4) [−2.7 to 6.8]	−1.2 (3.8) [−8.8 to 6.3]
OOP cost for comprehensive services			
No OOP cost	[Reference]	[Reference]	[Reference]
>0 Copayment	−4.6 (3.0) [−10.5 to 1.4]	−2.6 (2.8) [−8.0 to 2.9]	−14.6 (5.9) [−26.2 to −3.0]^c^
0% <Coinsurance <50%	−2.8 (4.5) [−11.7 to 6.0]	−2.6 (3.9) [−10.3 to 5.0]	4.3 (6.6) [−8.7 to 17.3]
≥ 50% Coinsurance	−3.7 (2.9) [−9.3 to 1.9]	−0.7 (2.8) [−6.2 to 4.7]	0.7 (5.0) [−9.1 to 10.5]
Preventive only benefit	12.1 (4.5) [3.2 to 21.0]^b^	7.8 (3.7) [0.6 to 15.0]^c^	−3.2 (4.8) [−12.6 to 6.1]
Annual benefit maximum			
0 <Benefit maximum ≤500	[Reference]	[Reference]	[Reference]
500 <Benefit maximum ≤1500	−4.8 (4.9) [−14.4 to 4.7]	−8.5 (4.5) [−17.4 to 0.4]^d^	11.1 (5.8) [−0.2 to 22.4]^d^
1500 <Benefit maximum ≤2000	−4.1 (5.7) [−15.2 to 7.0]	−6.2 (5.1) [−16.2 to 3.8]	10.5 (6.9) [−3.1 to 24.2]
2000 <Benefit maximum ≤2500	−5.9 (5.5) [−16.8 to 5.0]	−5.7 (5.1) [−15.8 to 4.3]	16.2 (7.5) [1.5 to 30.9]^c^
Benefit maximum >2500	−10.8 (5.5) [−21.6 to −0.0]^c^	−11.7 (4.7) [−20.9 to −2.4]^c^	21.6 (8.0) [6.0 to 37.3]^b^
No benefit maximum	−12.4 (4.4) [−20.9 to −3.8]^b^	−11.4 (4.1) [−19.5 to −3.3]^b^	12.4 (5.7) [1.2 to 23.6]^c^

Abbreviations: HMO, health maintenance organization; OOP, out of pocket; PPO, preferred provider organization.

^a^ All probit marginal effect estimates are weighted and consider the complex survey design of the Medicare Current Beneficiary Survey. Covariates included in regression models but not reported are age, sex, race and ethnicity, rurality, respondent household income, respondent educational attainment, job status, self−reported health status, census region, dual eligibility status, log of county median household income, county percent in poverty, population density, dentists per capita and Medicare Advantage penetration.

^b^ *P* < .01.

^c^ *P* < .05.

^d^ *P* < .10.

HMO dental plan enrollment was associated with greater unmet dental need due to cost (4.4 percentage points [95% CI, 0.9-7.8 percentage points]; *P* = .01) compared with enrollment in non-HMO plans. Compared with plans that did not have an OOP cost for comprehensive services, plans not covering those services were associated with unmet dental need due to cost (7.8 percentage points [95% CI, 0.6-15.0 percentage points]; *P* = .03). There was no substantial variation in reported unmet dental need due to cost among MA dental plan enrollees at different positive levels of coinsurance or copayment thresholds for comprehensive services. Relative to individuals in plans that imposed up to a $500 annual plan maximum, MA enrollees in plans with greater than $2500 annual plan maximum (−11.7 percentage points [95% CI, −20.9 to −2.4 percentage points]; *P* = .013) or no annual maximum (−11.4 percentage points [95% CI, −19.5 to −3.3 percentage points]; *P* = .006) reported lower rates of unmet dental need due to cost (Table 2). The estimated probability of an MA enrollee with dental benefits reporting an unmet dental need cost increased as annual plan maximums decreased (Figure 2B).

Relative to individuals enrolled in plans that did not have an OOP cost for comprehensive services, individuals enrolled in plans with a copay for comprehensive services had lower dental care use (−14.6 percentage points [95% CI, −26.2 to −3.0 percentage points]; *P* = .01). Relative to individuals in plans that imposed up to a $500 annual plan maximum, MA enrollees in dental plans that imposed between a $500 and $1500 annual maximum (11.1 percentage points [95% CI, −0.2 to 22.4 percentage points]; *P* = .06), between a $2000 and $2500 annual maximum (16.2 percentage points [95% CI, 1.5-30.9 percentage points]; *P* = .031),greater than $2500 annual plan maximum (21.6 percentage points [95% CI, 6.0-37.3 percentage points]; *P* = .007), or no annual maximum (12.4 percentage points [95% CI, 1.2-23.6 percentage points]; *P* = .03) had higher rates of dental care use (Table 2). The estimated probability of an MA enrollee with dental benefits reporting a dental visit increased as annual maximums increased (Figure 2C).

### Sensitivity Analyses

We performed a number of sensitivity analyses. First, we restricted our sample to MA enrollees enrolled in mandatory dental plans (ie, plans that do not require additional fees for enrollment) (eTable 3 in Supplement 1). Overall, the estimated marginal effect sizes for plan characteristics, OOP costs, and plan maximums were qualitatively similar to our main specification. In a second sensitivity analysis, we removed MA dental plan enrollees who switched between plans during the 12-month period. The estimated marginal effect sizes from this sensitivity analysis were also qualitatively similar to our main specification (eTable 4 in Supplement 1). In a final sensitivity analysis, we also removed individual and county-level covariates from our regression models. Overall, the results are qualitatively similar to our main specification (eTable 5 in Supplement 1).

## Discussion

To our knowledge, our study is the first to examine the association between dental plan attributes and dental care use among individuals enrolled in MA dental plans and their likelihood to report unmet dental needs and financial barriers to dental care. HMO dental plans were associated with higher rates of unmet dental need and unmet dental need due to cost. This may be due to HMO plans limiting care provision networks, although we were not able to explore this in our data.^17^ Future research should examine the breadth of dental care provision networks in MA dental plans and how this could affect access to dental care. We found that imposing OOP payments for preventive services and prior authorization requirements were associated with more unmet dental care needs. The resulting unmet care needs are consistent with broader findings that show MA plans use prior authorization to limit utilization and cost,^18^ which could limit access to care.^19^ Our findings may also lend support to the existing literature that shows MA dental benefits do little to enhance access to dental care.^10,11,12^

Enrollees in MA plans that did not cover comprehensive dental services had higher rates of unmet dental need and cost barriers, suggesting that preventive-only dental plans could hinder access to dental care. However, among plans that mandated OOP costs for comprehensive services, there was little variation in reported unmet dental need or unmet dental need due to cost at different levels of cost sharing. The finding with regard to coinsurance is inconsistent with broader research that finds a negative association of coinsurance rates with the likelihood of visiting a dentist.^20^ It is possible that plans with limited or no coinsurance control costs in ways that we were unable to observe in our data, such as using narrow care provision networks, for which we do not have data. As annual plan benefit maximums increased, we found that the likelihood of reporting unmet dental need was lower and visiting a dentist was higher. Further, our findings suggest that an annual plan benefit maximum of at least $2500 was associated with an increase in access to dental care.

### Limitations

Our study has several limitations. First, although we controlled for several individual and county-level characteristics, the observational study type does not allow causal links to be made between MA dental plan benefit design characteristics and measures of dental care access. Second, there were several potential confounders that we were unable to include in our regression models, such as measures of network adequacy, which are likely to be associated with benefit design attributes and measures of dental care access. Third, while most MA plans are managed by private dental insurers, our findings only applied to the MA population and were not generalizable to employer-sponsored dental benefits or the individual dental insurance market. Fourth, due to endogeneity from plan choice arising from enrollees with greater dental needs choosing more comprehensive plans, our estimates could overestimate the effect of plan generosity on dental care access. Lastly, we believe the CMS data, for some MA dental plans, do not effectively distinguish copays from coinsurance (eg, some MA dental plans have copayments for comprehensive services greater than $100, which is unusual). Due to this potential measurement error, the fact that our coinsurance regressor was not significant while the copay regressor was significant in our dental care use model should be interpreted with caution. Namely, it should not be grounds for concluding that copays matter but coinsurance does not.

## Conclusions

Results of this study suggest that dental plan offerings that attempt to limit resource utilization through HMOs or prior authorization requirements or that cover only preventive dental services are associated with barriers to dental care for MA enrollees. Annual plan benefit maximums below $2500 were associated with higher rates of unmet dental need and lower dental care utilization. These findings suggest that MA dental plans could be regulated to improve access to care among beneficiaries.

## Notes

Supplement 1. **eTable 1.** Reported Unmet Dental Need, Unmet Dental Need Due to Cost and Dental Care Utilization by Insurance Status **eTable 2.** Summary Statistics. Beneficiary and Local Area Characteristics **eTable 3.** Relationship of Unmet Dental Need, Unmet Dental Need Due to Cost and Dental Care Utilization to MA Dental Plan Attributes. Sub-Sample of MA Enrollees with Mandatory Dental Benefits **eTable 4.** Relationship of Unmet Dental Need, Unmet Dental Need Due to Cost and Dental Care Utilization to MA Dental Plan Attributes. Sub-Sample of MA Enrollees in the Same Dental Plan for 12 Months **eTable 5.** Relationship of Unmet Dental Need, Unmet Dental Need Due to Cost and Dental Care Utilization to MA Dental Plan Attributes. No Individual or County-Level Covariates

Supplement 2.
Data Sharing Statement
