# Supplementary material for: Benefit Design and Access to Dental Care Among Seniors With Medicare Advantage Dental Benefits
Source: JAMA Health Forum. 2025 Jan 24;6(1):e245123. doi: 10.1001/jamahealthforum.2024.5123 (PMC11762240; doi:10.1001/jamahealthforum.2024.5123)
Supplement: Supplement 1. — eTable 1. Reported Unmet Dental Need, Unmet Dental Need Due to Cost and Dental Care Utilization by Insurance Status eTable 2. Summary Statistics. Beneficiary and Local Area Characteristics eTable 3. Relationship of Unmet Dental Need, Unmet Dental Need Due to Cost and Dental Care Utilization to MA Dental Plan Attributes. Sub-Sample of MA Enrollees with Mandatory Dental Benefits eTable 4. Relationship of Unmet Dental Need, Unmet Dental Need Due to Cost and Dental Care Utilization to MA Dental Plan Attributes. Sub-Sample of MA Enrollees in the Same Dental Plan for 12 Months eTable 5. Relationship of Unmet Dental Need, Unmet Dental Need Due to Cost and Dental Care Utilization to MA Dental Plan Attributes. No Individual or County-Level Covariates [file jamahealthforum-e245123-s001.pdf]

Supplemental Online Content

Nasseh K, Singhal A, Vujicic M, Simon L. Benefit design and access to dental care among seniors with Medicare Advantage dental benefits. *JAMA Health Forum.* 2025;6(1):e245123. doi:10.1001/jamahealthforum.2024.5123

eTable 1. Reported Unmet Dental Need, Unmet Dental Need Due to Cost and Dental Care Utilization by Insurance Status

eTable 2. Summary Statistics. Beneficiary and Local Area Characteristics

eTable 3. Relationship Of Unmet Dental Need, Unmet Dental Need Due to Cost and Dental Care Utilization to MA Dental Plan Attributes. Sub-Sample of MA Enrollees with Mandatory Dental Benefits

eTable 4. Relationship Of Unmet Dental Need, Unmet Dental Need Due to Cost and Dental Care Utilization to MA Dental Plan Attributes. Sub-Sample of MA Enrollees in the Same Dental Plan for 12 Months

eTable 5. Relationship Of Unmet Dental Need, Unmet Dental Need Due to Cost and Dental Care Utilization to MA Dental Plan Attributes. No Individual or County-Level Covariates

This supplemental material has been provided by the authors to give readers additional information about their work.

© 2025 Nasseh K et al. *JAMA Health Forum.*

eTable 1. Reported Unmet Dental Need, Unmet Dental Need Due to Cost and Dental Care Utilization by Insurance Status

	Medicare Advantage with MA Dental Benefit	Medicare Advantage with No MA Dental Benefit	Traditional Medicare
Reported Unmet Dental Need	12.5%	6.0%	8.1%
	[10.5%-14.4%]	[4.3%-7.7%]	[6.9%-9.3%]
Reported Unmet Dental Need Due to Cost	9.4%	5.1%	6.1%
	[7.8%-11.0%]	[3.5%-6.7%]	[5.0%-7.1%]
Visited Dentist in Year	47.0%	62.1%	59.2%
	[44.4%-49.6%]	[59.0%-65.2%]	[57.4%-61.0%]

Notes: All estimates are weighted and consider the complex survey design of the MCBS. Respondents with MA dental benefits have at least 1 month of enrollment in a MA dental plan. MA-Medicare Advantage. 95% Confidence Intervals in brackets

© 2025 Nasseh K et al. *JAMA Health Forum*.

eTable2. Summary Statistics. Beneficiary and Local Area Characteristics

Beneficiary Characteristics	
Age	74.7
	(7.44)
Female	0.584
	(0.520)
Racial/Ethnic Identity	
White	0.678
	(0.493)
Black	0.156
	(0.383)
Asian	0.019
	(0.143)
Hispanic	0.134
	(0.360)
Other Race	0.013
	(0.120)
Income	
FPL≤100%	0.201
	(0.423)
100%<FPL≤135%	0.146
	(0.373)
135%<FPL≤200%	0.187
	(0.411)
FPL>200%	0.466
	(0.526)
Rural	0.132
	(0.358)
Census Region	
New England/Middle-Atlantic	0.149
	(0.376)
East North Central	0.148
	(0.375)
West North Central	0.076
	(0.279)
South Atlantic	0.229
	(0.443)
East South Central	0.074
	(0.276)
West South Central	0.095
	(0.310)
Mountain	0.087

© 2025 Nasseh K et al. *JAMA Health Forum.*

	(0.298)
Pacific	0.142
	(0.369)
Self or Spouse Working	0.127
	(0.352)
Educational Attainment	
Less Than High School	0.218
	(0.436)
High School	0.294
	(0.481)
Some College	0.279
	(0.473)
College	0.125
	(0.349)
Post-Graduate	0.084
	(0.293)
Dual Eligible for Medicare and Medicaid	0.255
	(0.460)
Self-Reported Good or Excellent Health	0.801
	(0.422)
Local Area County Characteristics	
MA Penetration	0.438
	(0.126)
Median Household Income	62300.53
	(16190.68)
Percent Poverty	0.133
	(0.048)
Population Density	1927.12
	(6711.28)
Dentists Per Capita (Per 100K)	58.41
	(11.64)
Number of Observations	1789

Notes: All estimates are weighted and consider the complex survey design of the MCBS. Standard deviation in parentheses.

© 2025 Nasseh K et al. *JAMA Health Forum*.

eTable3. Relationship Of Unmet Dental Need, Unmet Dental Need Due to Cost and Dental Care Utilization to MA Dental Plan Attributes. Sub-Sample of MA Enrollees with Mandatory Dental Benefits

Dependent Variable	Reported Unmet Dental Need	Reported Unmet Dental Need Due to Cost	Visited Dentist in Year
HMO Plan	0.075***	0.046**	-0.020
	(0.023)	(0.021)	(0.040)
	[0.031 - 0.120]	[0.005 - 0.087]	[-0.099 - 0.059]
Two or More Dental Cleanings Per Year	0.004	0.005	-0.055
	(0.040)	(0.038)	(0.051)
	[-0.074 - 0.082]	[-0.069 - 0.079]	[-0.156 - 0.045]
OOP for Preventive Services	0.050*	0.021	-0.011
	(0.026)	(0.023)	(0.041)
	[-0.002 - 0.101]	[-0.025 - 0.066]	[-0.091 - 0.069]
Referral Required	0.048	0.050	0.043
	(0.037)	(0.034)	(0.044)
	[-0.024 - 0.120]	[-0.017 - 0.117]	[-0.042 - 0.129]
Prior Authorization Required	0.061**	0.042**	-0.021
	(0.024)	(0.021)	(0.036)
	[0.014 - 0.109]	[0.001 - 0.082]	[-0.091 - 0.049]
All Dental Services Offered	0.049	0.035	0.004
	(0.034)	(0.029)	(0.042)
	[-0.016 - 0.115]	[-0.023 - 0.092]	[-0.079 - 0.087]
		OOP on Comprehensive Services	
Zero OOP	REF	REF	REF
	-	-	-
	-	-	-
>0 Copayment	-0.049	-0.036	-0.125*
	(0.038)	(0.033)	(0.071)
	[-0.124 - 0.026]	[-0.101 - 0.029]	[-0.264 - 0.014]
0%<Coinsurance<50%	-0.029	-0.019	0.072
	(0.078)	(0.064)	(0.096)
	[-0.181 - 0.124]	[-0.144 - 0.107]	[-0.116 - 0.259]

© 2025 Nasseh K et al. *JAMA Health Forum*.

≥50% Coinsurance	-0.037	0.005	-0.004
	(0.031)	(0.034)	(0.057)
	[-0.098 - 0.024]	[-0.061 - 0.071]	[-0.115 - 0.107]
Preventive Only Benefit	0.140***	0.080**	-0.012
	(0.048)	(0.039)	(0.052)
	[0.045 - 0.235]	[0.003 - 0.156]	[-0.113 - 0.089]
		Annual Benefit Maximum	
O<Benefit Maximum≤500	REF	REF	REF
	-	-	-
	-	-	-
500<Benefit Maximum≤1,500	-0.031	-0.084*	0.102*
	(0.052)	(0.046)	(0.059)
	[-0.133 - 0.071]	[-0.175 - 0.007]	[-0.014 - 0.219]
1,500<Benefit Maximum≤2,000	-0.028	-0.061	0.145*
	(0.064)	(0.055)	(0.076)
	[-0.153 - 0.097]	[-0.169 - 0.047]	[-0.004 - 0.293]
2,000<Benefit Maximum≤2,500	-0.053	-0.052	0.166**
	(0.060)	(0.054)	(0.078)
	[-0.170 - 0.065]	[-0.157 - 0.053]	[0.014 - 0.318]
Benefit Maximum>2,500	-0.102*	-0.115**	0.215***
	(0.059)	(0.048)	(0.083)
	[-0.217 - 0.013]	[-0.209 - -0.022]	[0.053 - 0.377]
No Benefit Maximum	-0.118**	-0.096**	0.122**
	(0.046)	(0.044)	(0.060)
	[-0.209 - -0.027]	[-0.181 - -0.011]	[0.005 - 0.240]
Observations	1187	1187	1466

Notes: All probit marginal effect estimates are weighted and consider the complex survey design of the Medicare Current Beneficiary Survey (MCBS). Covariates included in regression models but not reported are age, sex, race/ethnicity, rurality, respondent household income, respondent educational attainment, job status, self-reported health status, census region, dual eligibility status, log of county median household income, county percent in poverty, population density, dentists per capita and MA penetration. Standard errors in parentheses. 95% confidence intervals in brackets. *** p<0.01, ** p<0.05, * p<0.1.

© 2025 Nasseh K et al. *JAMA Health Forum*.

eTable4. Relationship Of Unmet Dental Need, Unmet Dental Need Due to Cost and Dental Care Utilization to MA Dental Plan Attributes. Sub-Sample of MA Enrollees in the Same Dental Plan for 12 Months

Dependent Variable	Reported Unmet Dental Need	Reported Unmet Dental Need Due to Cost	Visited Dentist in Year
HMO Plan	0.079***	0.047***	-0.022
	(0.019)	(0.018)	(0.037)
	[0.041 - 0.117]	[0.013 - 0.082]	[-0.094 - 0.049]
Two or More Dental Cleanings Per Year	0.014	-0.004	-0.051
	(0.033)	(0.032)	(0.047)
	[-0.050 - 0.078]	[-0.067 - 0.060]	[-0.143 - 0.041]
OOP for Preventive Services	0.050**	0.031*	-0.020
	(0.022)	(0.018)	(0.036)
	[0.007 - 0.094]	[-0.005 - 0.067]	[-0.090 - 0.050]
Referral Required	0.019	0.021	0.037
	(0.030)	(0.028)	(0.041)
	[-0.041 - 0.079]	[-0.033 - 0.076]	[-0.044 - 0.119]
Prior Authorization Required	0.055**	0.046**	-0.015
	(0.022)	(0.018)	(0.033)
	[0.012 - 0.098]	[0.010 - 0.081]	[-0.080 - 0.051]
All Dental Services Offered	0.027	0.017	-0.008
	(0.028)	(0.024)	(0.040)
	[-0.028 - 0.083]	[-0.030 - 0.065]	[-0.086 - 0.070]
		OOP on Comprehensive Services	
Zero OOP	REF	REF	REF
	-	-	-
	-	-	-
>0 Copayment	-0.038	-0.013	-0.139**
	(0.033)	(0.032)	(0.061)
	[-0.103 - 0.026]	[-0.076 - 0.051]	[-0.258 - -0.019]
0%<Coinsurance<50%	-0.013	-0.012	0.046
	(0.050)	(0.045)	(0.067)
	[-0.111 - 0.084]	[-0.100 - 0.076]	[-0.086 - 0.178]
≥50% Coinsurance	-0.052*	-0.030	0.025
	(0.028)	(0.024)	(0.051)

© 2025 Nasseh K et al. *JAMA Health Forum*.

	[-0.106 - 0.003]	[-0.077 - 0.017]	[-0.076 - 0.125]
Preventive Only Benefit	0.130***	0.098**	-0.019
	(0.050)	(0.043)	(0.051)
	[0.031 - 0.228]	[0.015 - 0.182]	[-0.119 - 0.081]
		Annual Benefit Maximum	
0<Benefit Maximum≤500	REF	REF	REF
	-	-	-
	-	-	-
500<Benefit Maximum≤1,500	-0.041	-0.074	0.097
	(0.049)	(0.045)	(0.060)
	[-0.136 - 0.055]	[-0.163 - 0.014]	[-0.020 - 0.214]
1,500<Benefit Maximum≤2,000	-0.026	-0.042	0.106
	(0.057)	(0.052)	(0.072)
	[-0.139 - 0.087]	[-0.145 - 0.060]	[-0.035 - 0.247]
2,000<Benefit Maximum≤2,500	-0.058	-0.043	0.152**
	(0.054)	(0.051)	(0.077)
	[-0.165 - 0.048]	[-0.143 - 0.056]	[0.002 - 0.303]
Benefit Maximum>2,500	-0.092	-0.098**	0.198**
	(0.056)	(0.049)	(0.083)
	[-0.202 - 0.018]	[-0.194 - -0.003]	[0.035 - 0.361]
No Benefit Maximum	-0.121***	-0.114***	0.121**
	(0.043)	(0.040)	(0.059)
	[-0.206 - -0.036]	[-0.193 - -0.035]	[0.005 - 0.237]
Observations	1399	1399	1720

Notes: All probit marginal effect estimates are weighted and consider the complex survey design of the Medicare Current Beneficiary Survey (MCBS). Covariates included in regression models but not reported are age, sex, race/ethnicity, rurality, respondent household income, respondent educational attainment, job status, self-reported health status, census region, dual eligibility status, log of county median household income, county percent in poverty, population density, dentists per capita and MA penetration. Standard errors in parentheses. 95% confidence intervals in brackets. *** p<0.01, ** p<0.05, * p<0.1.

© 2025 Nasseh K et al. *JAMA Health Forum*.

eTable5. Relationship Of Unmet Dental Need, Unmet Dental Need Due to Cost and Dental Care Utilization to MA Dental Plan Attributes. No Individual or County-Level Covariates.

Dependent Variable	Reported Unmet Dental Need	Reported Unmet Dental Need Due to Cost	Visited Dentist in Year
HMO Plan	0.040*	0.013	-0.003
	(0.021)	(0.019)	(0.035)
	[-0.002 - 0.082]	[-0.025 - 0.052]	[-0.071 - 0.065]
Two or More Dental Cleanings Per Year	0.030	0.019	-0.078*
	(0.029)	(0.027)	(0.046)
	[-0.027 - 0.087]	[-0.034 - 0.072]	[-0.169 - 0.012]
OOP for Preventive Services	0.062***	0.045**	-0.042
	(0.022)	(0.018)	(0.036)
	[0.020 - 0.104]	[0.010 - 0.080]	[-0.112 - 0.028]
Referral Required	0.038	0.023	0.080**
	(0.030)	(0.024)	(0.038)
	[-0.022 - 0.097]	[-0.024 - 0.070]	[0.005 - 0.155]
Prior Authorization Required	0.052**	0.040**	-0.056*
	(0.022)	(0.019)	(0.033)
	[0.009 - 0.096]	[0.003 - 0.077]	[-0.120 - 0.008]
All Dental Services Offered	0.008	0.002	0.045
	(0.025)	(0.022)	(0.036)
	[-0.042 - 0.057]	[-0.042 - 0.045]	[-0.026 - 0.116]
		OOP on Comprehensive Services	
Zero OOP	REF	REF	REF
	-	-	-
	-	-	-
>0 Copayment	-0.075**	-0.034	-0.046
	(0.033)	(0.032)	(0.054)
	[-0.140 - -0.009]	[-0.096 - 0.028]	[-0.153 - 0.060]
0%<Coinsurance<50%	-0.094***	-0.065**	0.153**
	(0.033)	(0.029)	(0.067)
	[-0.159 - -0.029]	[-0.123 - -0.008]	[0.022 - 0.285]
≥50% Coinsurance	-0.076**	-0.044*	0.101**
	(0.030)	(0.025)	(0.049)
	[-0.135 - -0.017]	[-0.094 - 0.005]	[0.004 - 0.198]
Preventive Only Benefit	0.066	0.035	-0.006
	(0.048)	(0.035)	(0.048)
	[-0.028 - 0.159]	[-0.035 - 0.104]	[-0.099 - 0.087]
		Annual Benefit Maximum	
0<Benefit Maximum≤500	REF	REF	REF

© 2025 Nasseh K et al. *JAMA Health Forum.*

	-	-	-
	-	-	-
500<Benefit Maximum≤1,500	0.002	-0.046	0.048
	(0.050)	(0.043)	(0.059)
	[-0.097 - 0.101]	[-0.131 - 0.039]	[-0.068 - 0.164]
1500<Benefit Maximum<=2000	-0.012	-0.033	0.072
	(0.058)	(0.052)	(0.069)
	[-0.125 - 0.101]	[-0.134 - 0.069]	[-0.063 - 0.207]
2000<Benefit Maximum<=2500	-0.031	-0.035	0.192***
	(0.056)	(0.051)	(0.074)
	[-0.141 - 0.079]	[-0.135 - 0.065]	[0.047 - 0.337]
Benefit Maximum>2500	-0.075	-0.076	0.205**
	(0.057)	(0.053)	(0.085)
	[-0.187 - 0.038]	[-0.180 - 0.027]	[0.039 - 0.371]
No Benefit Maximum	-0.094**	-0.095**	0.149***
	(0.043)	(0.038)	(0.057)
	[-0.177 - -0.010]	[-0.170 - -0.020]	[0.038 - 0.259]
Observations	1455	1455	1789

Notes: All estimates are weighted and consider the complex survey design of the MCBS. Standard errors in parentheses. 95% confidence intervals in brackets. *** p<0.01, ** p<0.05, * p<0.1.

© 2025 Nasseh K et al. *JAMA Health Forum*.
